# Trends in respiratory diseases before and after the COVID-19 pandemic in China from 2010 to 2021

**DOI:** 10.1186/s12889-023-15081-4

**Published:** 2023-02-01

**Authors:** Zhongbao Zuo, Chunli Yang, Fei Ye, Miaochan Wang, Jing Wu, Chengjiang Tao, Yunhao Xun, Zhaoyi Li, Shourong Liu, Jinsong Huang, Aifang Xu

**Affiliations:** 1 Department of Clinical Laboratory, Hangzhou Xixi Hospital, 2 Hengbu Road, Xihu District, Zhejiang, 310023 China; 2 Department of Clinical Laboratory, The 903rd Hospital of PLA, Zhejiang, 310013 China; 3 Health Examination Center, Hangzhou Xixi Hospital, Zhejiang, 310023 China; 4 Department of Obstetrics and Gynecology, Hangzhou Xixi Hospital, Zhejiang, 310023 China; 5 Department of Hepatology, Hangzhou Xixi Hospital, Zhejiang, 310023 China; 6 Science and Education Department, Hangzhou Xixi Hospital, Zhejiang, 310023 China

**Keywords:** Respiratory diseases, COVID-19, Nonpharmaceutical interventions, Seasonal influenza, Epidemiology

## Abstract

**Background:**

The ongoing benefits of coronavirus disease 2019 (COVID-19) nonpharmaceutical interventions (NPIs) for respiratory infectious diseases in China are still unclear. We aimed to explore the changes in seven respiratory infectious diseases before, during, and after COVID-19 in China from 2010 to 2021.

**Methods:**

The monthly case numbers of seven respiratory infectious diseases were extracted to construct autoregressive integrated moving average (ARIMA) models. Eight indicators of NPIs were chosen from the COVID-19 Government Response Tracker system. The monthly case numbers of the respiratory diseases and the eight indicators were used to establish the Multivariable generalized linear model (GLM) to calculate the incidence rate ratios (IRRs).

**Results:**

Compared with the year 2019, the percentage changes in 2020 and 2021 were all below 100% ranging from 3.81 to 84.71%. Pertussis and Scarlet fever started to increase in 2021 compared with 2020, with a percentage change of 183.46 and 171.49%. The ARIMA model showed a good fit, and the predicted data fitted well with the actual data from 2010 to 2019, but the predicted data was bigger than the actual number in 2020 and 2021. All eight indicators could negatively affect the incidence of respiratory diseases. The seven respiratory diseases were significantly reduced during the COVID-19 pandemic in 2020 and 2021 compared with 2019, with significant estimated IRRs ranging from 0.06 to 0.85. In the GLM using data for the year 2020 and 2021, the IRRs were not significant after adjusting for the eight indicators in multivariate analysis.

**Conclusion:**

Our study demonstrated the incidence of the seven respiratory diseases decreased rapidly during the COVID-19 pandemic in 2020 and 2021. At the end of 2021, we did see a rising trend for the seven respiratory diseases compared to the year 2020 when the NPIs relaxed in China, but the rising trend was not significant after adjusting for the NPIs indicators. Our study showed that NPIs have an effect on respiratory diseases, but Relaxation of NPIs might lead to the resurgence of respiratory diseases.

**Supplementary Information:**

The online version contains supplementary material available at 10.1186/s12889-023-15081-4.

## Introduction

The first confirmed Chinese case of COVID-19 was reported in December 2019 [1, 2], causing the pandemic around the country. COVID-19 subsequently swept the world [3–6], and the number of COVID-19 cases has exceeded 528 million worldwide, involving more than 200 countries [7]. China has implemented public interventions to prevent the pandemic, including both pharmaceutical measures (vaccines, antibodies, ventilators, etc.) and nonpharmaceutical measures (wear masks, travel restrictions, school closing, cancel public events, stay at home requirements, etc.). Although pharmaceutical measures can target specific pathogens, nonpharmaceutical interventions affect a wide range of infectious diseases. Recent studies [8–10] have shown that the nonpharmaceutical interventions (NPIs) (lockdown, restriction on gathering, etc.) during the COVID-19 pandemic may reduce the prevalence of sexually transmitted diseases. Similar outcomes were also seen in Tuberculosis [11], seasonal influenza [12], and some other diseases [11, 13, 14]. 40 notifiable diseases need to be monitored, and some of them such as HBV [15], and Tuberculosis [16] had millions of new cases every year. We should pay attention to other infectious diseases in the context of COVID-19.

Some studies [11–14, 17] explored the impact of nonpharmaceutical interventions on respiratory infectious diseases during the COVID-19 pandemic. However, most studies only focused on the year 2020 when the COVID-19 outbroke. The ongoing NPIs persisted for years but changed with the level when the pandemic relaxed. The long-term ongoingnonpharmaceutical interventions for respiratory infectious diseases in China are still unclear, and there are still no studies based on national annual data. In this study, we aimed to explore the changes in seven respiratory infectious diseases before, during, and after COVID-19 in the Chinese mainland from 2010 to 2021.

## Method

### Data collection

The monthly case numbers of seven respiratory infectious diseases (Measles, Tuberculosis, Pertussis, Scarlet fever, Seasonal Influenza, Mumps, Rubella) were extracted from the website of the National Health Commission (http://www.nhc.gov.cn/jkj/s2907/new_list.shtml). All the data published by the Commission were originally from the China Information System for Disease Control and Prevention (CISDCP) [18, 19], which was a real-time disease-reporting system covering 40 notifiable infectious diseases (COVID-19 included in January 2020). The surveillance system was first established in 2004, covering 397 cities in 31 provinces in mainland China, covering a population of about 1.4 billion people. This system is based on the network and operates through administrative hierarchical responsibility and geographical management. The clinicians fill in the standard case report card for respiratory infectious diseases. Epidemic reports are time-sensitive, and respiratory infectious diseases should be reported within 24 h. All notifiable infectious diseases were timely reported to the local centers for disease control and prevention after diagnosis according to their standard criteria [20]. Seven respiratory infectious diseases without detailed information from the government’s website were included in our research. Demographic statistics data came from the website of the statistical yearbook of the National Bureau of Statistics (http://www.stats.gov.cn/tjsj/ndsj/). The study was approved by the institutional ethics review committee at Hangzhou Xixi Hospital (2022 Science Ethic No.36). Waiver of informed consent was granted by the institutional ethics review committee at Hangzhou Xixi Hospital (2022 Science Ethic No.36).

The confirmed Chinese COVID-19 cases in 2020 and 2021 came from the 2019 Novel Coronavirus COVID-19 (2019-nCoV) Data Repository provided by Johns Hopkins University [21]. The implementation of the main NPIs in China from 2020 to 2021 is illustrated in the supplementary Fig. 1. The indicators of government control measures were extracted from COVID-19 Government Response Tracker (GRT) [22]. Eight indicators were chosen from the GRT system, which were workplace closing, school closing, cancel public events, stay-at-home requirements, restrictions on gathering size, closed public transport, restrictions on internal movement, and international travel controls. The strictness of the nonpharmaceutical interventions increased with the score of indicators. The indicators are recorded on an ordinal scale that represents the level of strictness of the policy. Government coronavirus policies often vary by region within countries. Coding the most stringent government policy that is in place in a country/territory, as represented by the highest ordinal value. Sometimes the most stringent policy in a country/territory will only apply to a small part of the population. If the most stringent policy is only present in a limited geographic area or sector (e.g. perhaps only one province has implemented policies at a high level), the indicators use a binary flag variable to denote this limited scope. The detailed method of the NPIs can be found on the website (https://www.bsg.ox.ac.uk/research/research-projects/covid-19-government-response-tracker).

### Statistical analysis

#### Percentage change analysis

The pandemic phases were defined as Phase 1 (2010–2018, before COVID-19), Phase 2 (2019, pervious year of COVID-19), Phase 3 (2020, first year of COVID-19), and Phase 4 (2021, second year of COVID-19). Here we calculated the percentage change of (2010–2018)/2019 with the average yearly cases of 2010–2018 divided by the reported cases of the year 2019, 2020/2019 was the number of the year 2020 divided by the number of the year 2019, and so on 2021/2019 and 2021/2020.

### Autoregressive integrated moving average (ARIMA) model construction

ARIMA models were used to formulate the trend of respiratory diseases with the monthly data, and we used the forecast::auto.Arima() function in the R software to find a fitted model. Root mean square error (RMSE), mean absolute percentage error (MAPE), Akaike’s information criterion (AIC), and Bayesian information criterion (BIC) were used to evaluate the goodness-of-fit of constructed models. Ljung–The box test was used to check whether the residual of the model was a white noise (*p* > 0.05 for white noise). To estimate the benefits of NPIs against COVID-19 on respiratory diseases, we used the relative reduction as the indicator, and the calculation formula of relative reduction was: Relative reduction (%) = 100% × (number of expected cases – number of observed cases)/number of expected cases.

### Correlation analysis

The daily national indicators of the eight COVID-19 control measures from the GRT system were newly calculated by the 31 provinces’ average score, and the monthly national indicators were the average of the daily data. The Pearson correlation analysis was conducted to measure the relationship between the monthly eight indicators and monthly COVID-19 cases and seven respiratory diseases. A two-sided *P* < 0.05 was considered statistically significant.

### Multivariable generalized linear model construction

To adjust potential confounding factors of NPI, e.g., long-term disease trends, and indicators of government control measures, some multivariable generalized linear models (GLM) were constructed to explore the impact of NPIs on each respiratory disease. The pandemic phases were defined as Phase 1 (2010–2018), Phase 2 (2019), Phase 3 (2020), and Phase 4 (2021), and the reference period is Phase 2 (2019). X-13ARIMA-SEATS (signal extraction) method was used to obtain seasonality-removed monthly case numbers for each disease [13]. SEATS decomposes the time series into seasonal, trend, transitory, and irregular components, assigning deterministic effects to each component. A fundamental assumption made by SEATS is that the linearized time series ${y_t}$ (log of monthly case numbers in our analysis) follows the ARIMA model

$$\AE\left(\mathrm B\right)\mathbf F\left({\mathrm B}_{\mathrm s}\right)\left(\mathbf1-\mathrm B\right)^{\mathrm d}\left(\mathbf1-{\mathrm B}_{\mathrm s}\right)^{\mathrm D}\left(y_t-x_t^l\;\beta\right)=\theta\left(B\right)\circleddash\left(B_s\right)\alpha_{\mathrm t}$$

${y_t}$ is the time series, $x_{t}^{'}\beta$ is the regression part with covariates $xt$, $\alpha \text{t}$ is the white noise with mean 0 and variance σ,

*B* and $B\text{s}$: the non-seasonal and seasonal operators, *B *(${y_t}$)=$yt - 1$, $B\text{s}$(${y_t}$)=$yt - 12$;

$\AE\left(B\right)=1-AE1\;B^1-\dots\;-AE\;\rho B^\rho$, reflects a non-seasonal autoregressive (AR) operator of order ρ;

$\mathrm F\left({\mathrm B}_{\mathrm s}\right)=1-\;{\mathrm{AE}}_1\;{\mathrm B}_{\mathrm s}^1-\dots-\;\AE\;\mathrm\rho B_{\mathrm s}^\rho$ , reflects seasonal AR operator of order ρ;

$\mathbf{\left({1\mathrm-\mathrm B}\right)}^{\mathrm d}\mathbf{\left({1\mathrm-{\mathrm B}_{\mathrm s}}\right)}^{\mathrm D}$, non-seasonal and seasonal operators of orders d and D;

$\theta(B)=1-/AE_1\mathbf{\left(\mathrm B\right)}^1-\dots-/AE_q\left(B\right)^{\mathrm q}$, reflects non-seasonal moving average (MA) order of q;

$\Theta (B_\text{s})$= 1-$\Theta_1(B_\text{s}{)^1}$-…-$\Theta_\text{Q}(B_\text{s}{)^Q}$, reflects seasonal MA order of Q.

The holiday effect of the Chinese New Year was adjusted by the “genhol” function in the R package “seasonal” (version 1.8.3). SEATS automatically detects the shifts in the mean level of the time series, which means it can partially account for the impact of NPIs during the COVID-19 pandemic when estimating seasonality. Two outputs were obtained from SEATS, the seasonality-removed monthly case numbers and the seasonal trend itself. Using the seasonality-removed monthly case numbers to construct GLM with two stages. In stage I, we fitted GLM with the quasi-Poisson method using the following factors: phase 1–4 indicators for the year 2010–2021, long-term trend, number of person-days (the number of days times population size) as an offset. In stage II, we extracted the residuals from the stage I model with one month lag as an independent variable to account for autocorrelation. The incidence rate ratios (IRR) estimated by the model of stage II reflect the effects of COVID-19 NPIs on the incidence of seven respiratory diseases in 2020 and 2021 [23]. In addition, we selected the pandemic year of 2020 and 2021 to construct a GLM, with eight factors extracted from the GRT system added to the model in stage I. Stage II also included the residuals from the stage I model with one month lag, and the IRR of 2021 compared with 2020 would reflect the effects of COVID-19 NPIs. A sensitivity analysis using the harmonic functions to adjust for seasonality (detailed in the Supplementary material) was conducted in this research. All statistical analyses were conducted in R software (version 4.0.5, R Development Core Team 2020). Two-sided *P* < 0.05 was considered statistically significant.

## Results

### Percentage change of the seven respiratory diseases before and after COVID-19

**Fig. 1:**
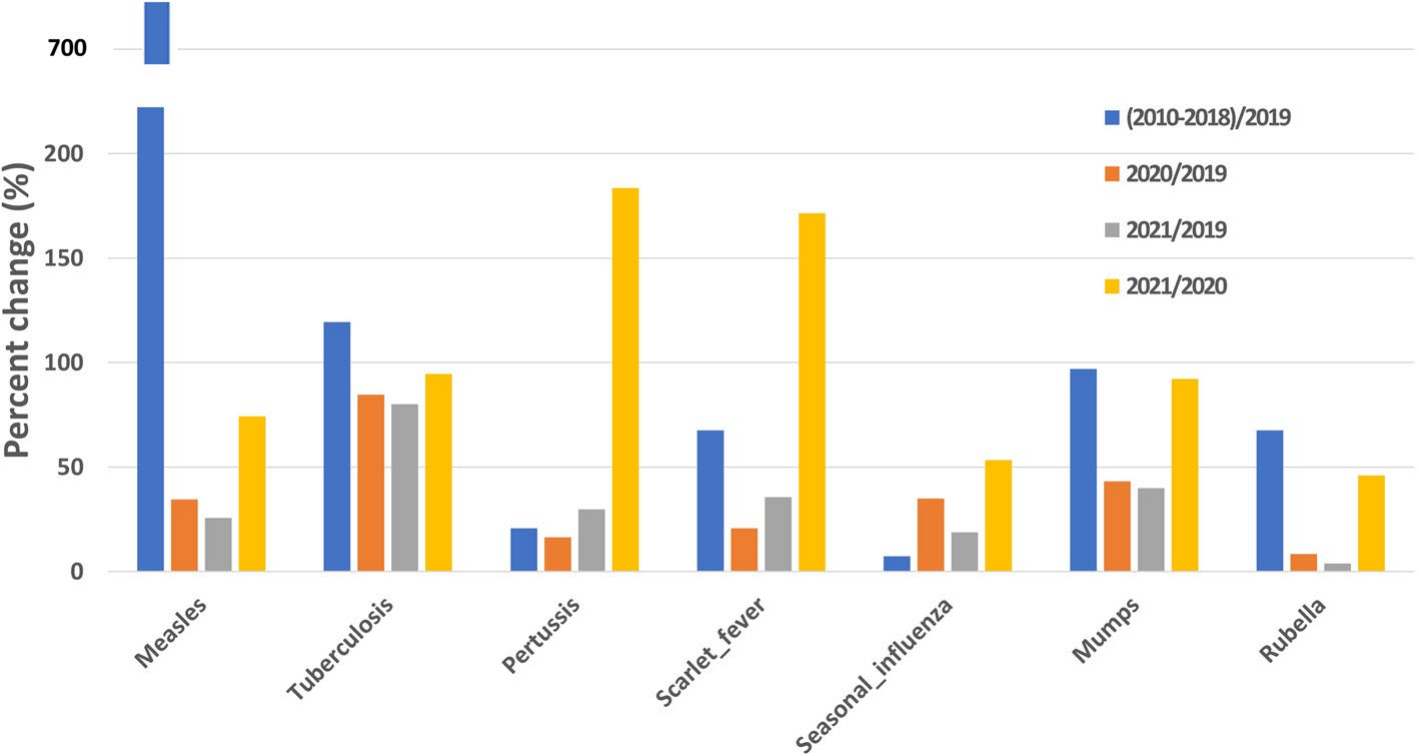
Percentage change of seven respiratory diseases before and after COVID-19. Percentage change of (2010–2018)/2019 was calculated with the average yearly cases of 2010–2018 divided by the number of the year 2019, 2020/2019 was the number of the year 2020 divided by the number of the year 2019, and so on 2021/2019 and 2021/2020

There were 25,980,811 incident cases included in our research, with a yearly average of 2,165,068 cases which was a huge burden on the public health of China. Compared with the year 2019, the percentage changes in 2020 and 2021 were all below 100% ranging from 3.81 to 84.71% (Table 1, Fig. 1). Furthermore, the percentage changes of 2020/2019 and 2021/2019 for Measles, Pertussis, Scarlet fever, Seasonal influenza, Mumps, and Rubella were all below 50%, indicating a more than 50% reduction of the six respiratory diseases during the COVID-19 era compared with the year 2019. Pertussis and Scarlet fever started to increase in 2021 compared with 2020, with a percentage change of 183.46 and 171.49%, but cases of year 2021 for the Measles, Tuberculosis, Seasonal influenza, Mumps, and Rubella remained lower than the reported cases of the year 2020.

**Table 1:** The percentage change of seven respiratory diseases before and after COVID-19

Disease	Percent change (%)				
	(2010–2018)/2019	2020/2019	2021/2019	2021/2020	(2020–2021)/ (2010–2019)
Measles	722.26	34.54	25.64	74.23	4.12
Tuberculosis	119.41	84.71	80.03	94.47	66.96
Pertussis	20.76	16.25	29.82	183.46	72.89
Scarlet fever	67.50	20.72	35.54	171.49	33.99
Seasonal influenza	7.34	34.98	18.67	53.36	65.71
Mumps	96.83	43.19	39.85	92.26	39.59
Rubella	67.44	8.31	3.81	45.86	6.06

Percentage change of (2010–2018)/2019 was calculated with the average yearly cases of 2010–2018 divided by the number of the year 2019, 2020/2019 was the number of the year 2020 divided by the number of the year 2019, and so on 2021/2019, 2021/2020 and (2020–2021)/ (2010–2019)

### ARIMA model construction and forecast

**Fig. 2:**
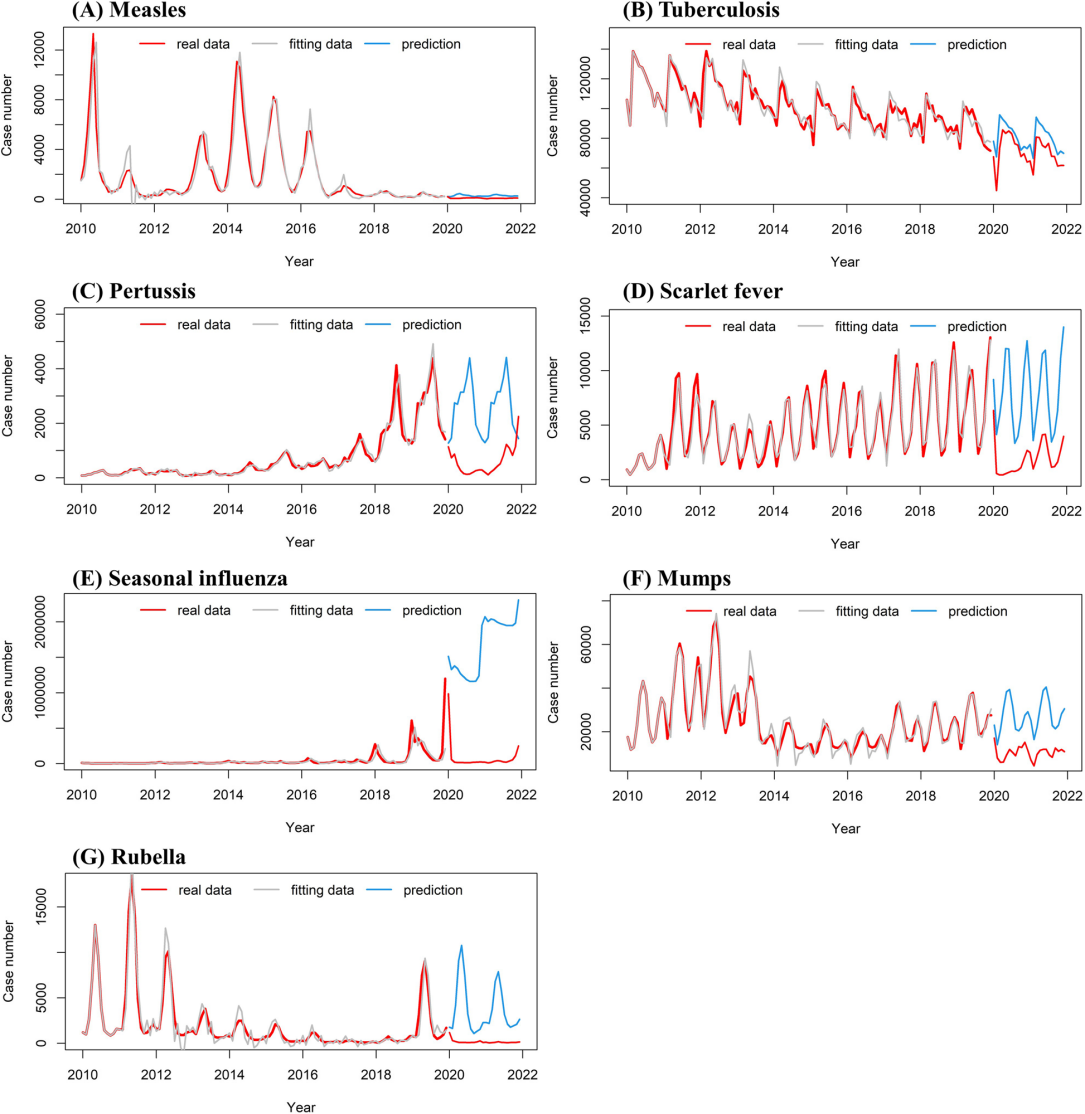
Autoregressive integrated moving average model fitting and prediction of the reported case numbers of seven respiratory diseases. **A** Measles, **B** Tuberculosis, **C** Pertussis, **D** Scarlet fever, **E** Seasonal influenza, **F** Mumps, **G** Rubella

The optimal models for the seven respiratory diseases were listed in Table 2. The MAPE showed a good fit of the models, and all the models passed the Ljung-Box test. The predicted data fitted well with the actual data from 2010 to 2019, but the predicted data was bigger than the actual number (Fig. 2) in 2020 and 2021 corresponding to the COVID-19. The trend of Mumps, Rubella, and Tuberculosis showed the same pace in 2020 and 2021, but Pertussis, Scarlet fever, and Seasonal influenza started to rise at the end of 2021. Tuberculosis has the smallest relative reduction, but Seasonal influenza and Rubella have the highest relative reduction. The detail of the relative reduction for the seven respiratory diseases can be seen in the Supplementary Table S1.

**Table 2:** Parameters and goodness-of-fit of the seven respiratory diseases’ optimal ARIMA models

Disease	Optimal model	Goodness of fit				Ljung-Box test	
		RMSE	MAPE (%)	AIC	BIC	χ^2^ value	*P*-value
Measles	(0,1,1) x (1,0,0)_12_	1019.25	31.44	1999.4	2007.74	< 0.001	0.99
Tuberculosis	(3,0,0) x (0,1,1)_12_	5636.78	3.90	2204.21	2220.3	0.013	0.91
Pertussis	(0,1,0) x (0,1,0)_12_	213.21	14.87	1465.45	1468.12	0.804	0.37
Scarlet fever	(1,0,1) x (0,1,2)_12_	814.56	15.09	1789.4	1805.5	0.096	0.76
Seasonal influenza	(0,1,0) x (0,0,2)_12_	102,523.5	39.97	3097.5	3105.84	0.076	0.78
Mumps	(2,1,1) x (0,1,2)_12_	3355.88	11.55	2073.05	2089.09	0.180	0.67
Rubella	(3,1,1) x (2,1,0)_12_	883.19	64.38	1788.69	1807.4	0.032	0.86

*RMSE* root mean square error, *MAPE* mean absolute percentage error, *AIC* Akaike’s information criterion, *BIC* Bayesian information criterion

### Correlation analysis for the eight indicators and the COVID-19 cases, seven respiratory diseases

The monthly number of COVID-19 cases was only negatively associated with Tuberculosis (*r* = − 0.57, *P* < 0.01) (Table 3). All the eight government control measures could affect the incidence of respiratory diseases. Close public transport was only negatively correlated with Scarlet fever (*r* = − 0.59, *P* < 0.01), and Stay at home was negatively correlated with Scarlet fever (*r* = − 0.51, *P* < 0.05) and Mumps (*r* = − 0.44, *P* < 0.05) (Table 3). School closing and Workplace closing were simultaneously negatively associated with Measles (*r* = − 0.50/− 0.52, *P* < 0.05/0.05), Scarlet fever (*r* = − 0.53/− 0.55, *P* < 0.05/0.01), and Mumps (*r* = − 0.61/− 0.59, *P* < 0.01/0.01), respectively. Except for the Scarlet fever, Restrictions on internal movement had a negative correlation with the other six respiratory diseases. In addition, Cancel public events, Restrictions on gatherings, and International travel controls were negatively correlated with 5 (Measles, Scarlet fever, Seasonal influenza, Mumps, and Rubella), 4 (Measles, Tuberculosis, Scarlet fever, and Mumps), and 4 (Tuberculosis, Scarlet fever, Seasonal influenza, and Rubella) respiratory diseases (Table 3), respectively.

**Table 3:** The correlation coefficients (r) of seven Respiratory infectious diseases with the COVID-19 case numbers and control measures in 2020 and 2021

Variable	Measles	Tuberculosis	Pertussis	Scarlet fever	Seasonal influenza	Mumps	Rubella
School closing	−0.50^*^	−0.21	0.12	− 0.53^*^	− 0.33	− 0.61^**^	− 0.22
Workplace closing	− 0.52^*^	− 0.18	0.23	− 0.55^**^	− 0.33	−0.59^**^	− 0.28
Cancel public events	−0.66^**^	− 0.30	0.12	− 0.61^**^	−0.49^*^	− 0.74^**^	−0.44^*^
Restrictions on gatherings	−0.56^**^	−0.49^*^	0.20	−0.48^*^	− 0.29	−0.71^**^	− 0.24
Close public transport	−0.11	− 0.2	−0.07	− 0.59^**^	−0.24	− 0.31	−0.08
Stay at home	−0.30	−0.39	0.06	−0.51^*^	− 0.24	−0.44^*^	− 0.10
Restrictions on internal movement	−0.51^*^	− 0.48^*^	0.54^*^	**− 0.30**	−0.41^*^	− 0.42^*^	−0.51^*^
International travel controls	−0.38	0.58^**^	−0.37	−0.46^*^	− 0.66^**^	−0.24	− 0.75^**^
COVID-19 cases	−0.08	− 0.57^**^	0.11	− 0.05	0.09	− 0.12	0.24

* *P* < 0.05, display in bolded font;

** *P* < 0.01, display in bolded italics font

### Generalized linear model-estimated association of NPIs with disease trend

All the seven respiratory diseases were significantly reduced during the COVID-19 pandemic in phase 3 (2020) and phase 4 (2021) compared with 2019, with significant estimated incidence rate ratios (IRRs) ranging from 0.06 to 0.85 (Table 4). For phase 1 (2010–2018), the significant IRRs of Measles and Tuberculosis were 4.69 (3.35–6.56) and 1.22 (1.16–1.28), but Pertussis, Scarlet fever, Seasonal influenza, and Rubella were below 1 with *P*-value < 0.001 (Table 4). The sensitivity analysis using harmonic functions showed qualitatively similar to the primary analysis (Supplementary Table S2).

**Table 4:** Model-estimated incidence rate ratio (IRR) of seven Respiratory infectious diseases

Disease	Phase 2 (2019) reported cases	Phase 1(2010–2018)		Phase 3 (2020)		Phase 4 (2021)	
		IRR (95%CI)	*P-value*	IRR (95%CI)	*P-value*	IRR (95%CI)	*P-value*
Measles	3573	4.69 (3.35–6.56)	< 0.001	**0.35 (0.19–0.67)**	**0.002**	**0.28 (0.14–0.57)**	**0.001**
Tuberculosis	1,034,760	1.22 (1.16–1.28)	< 0.001	**0.85 (0.79–0.91)**	**< 0.001**	**0.80 (0.75–0.85)**	**< 0.001**
Pertussis	30,727	**0.21 (0.18–0.25)**	**< 0.001**	**0.20 (0.15–0.28)**	**< 0.001**	**0.36 (0.27–0.48)**	**< 0.001**
Scarlet fever	83,028	**0.70 (0.61–0.81)**	**< 0.001**	**0.18 (0.13–0.25)**	**< 0.001**	**0.37 (0.29–0.47)**	**< 0.001**
Seasonal influenza	3,507,306	**0.09 (0.07–0.12)**	**< 0.001**	**0.20 (0.13–0.33)**	**< 0.001**	**0.26 (0.16–0.40)**	**< 0.001**
Mumps	303,105	**0.94 (0.88–1.01)**	**0.121**	**0.44 (0.38–0.49)**	**< 0.001**	**0.41 (0.36–0.47)**	**< 0.001**
Rubella	34,151	**0.56 (0.46–0.69)**	**< 0.001**	**0.08 (0.05–0.15)**	**< 0.001**	**0.06 (0.02–0.14)**	**< 0.001**

Generalized linear models (GLM) were used for estimating the IRRs of seven Respiratory infectious diseases. The seasonality of the reported cases was removed by a time series method in this model. IRR < 1 with *P* < 0.05 indicates a significant decline in incidence rate in the year 2020 compared to the year 2019. All *p*-values are two-sided and not adjusted for multiple comparisons. The reference period is the year 2019. Statistically significant reductions (IRR < 1) are displayed in bolded font

**Fig. 3:**
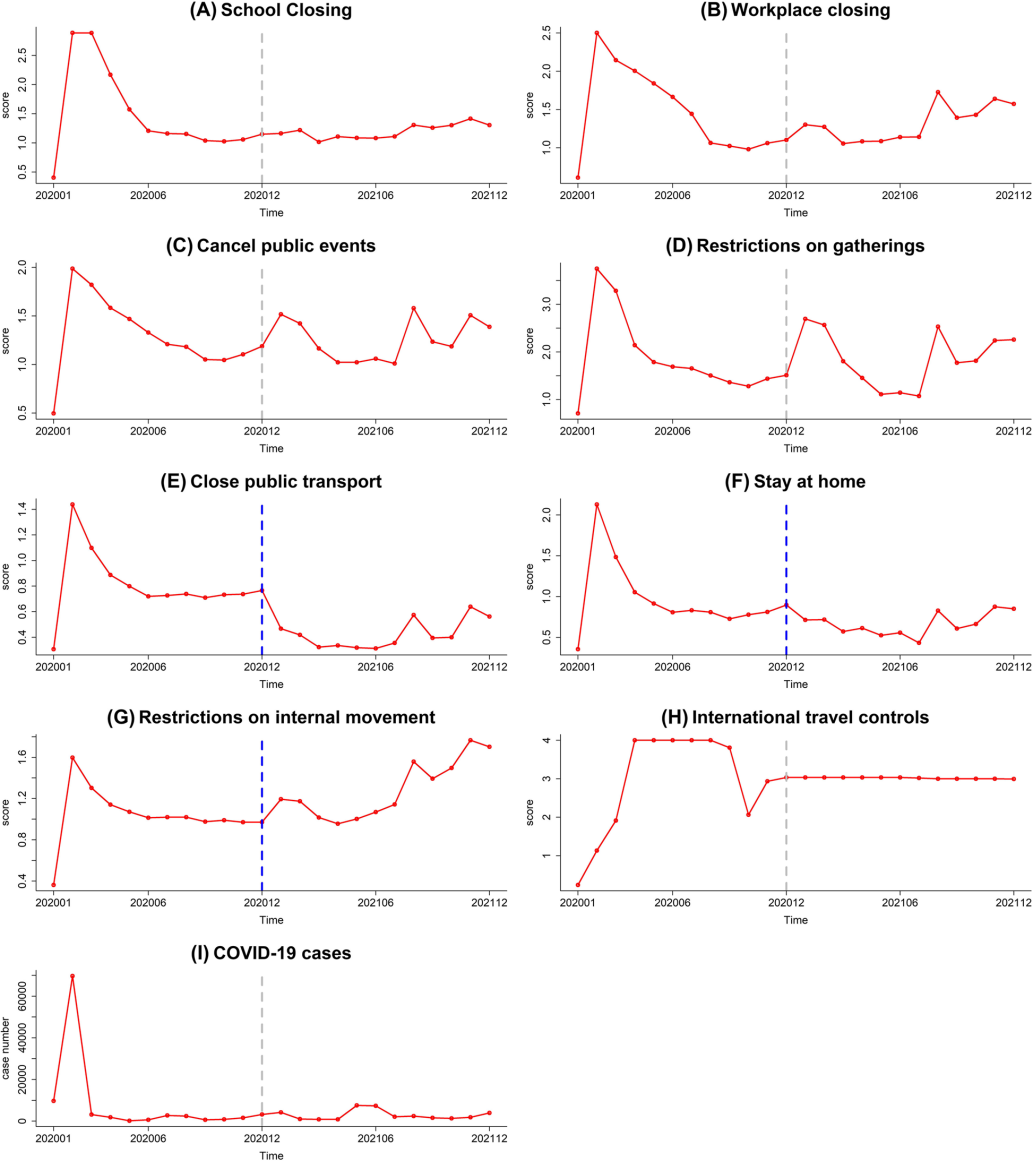
Scores of eight COVID-19 control measures and trends of COVID-19 case numbers in 2020 and 2021. Grey dashed lines are not statistically significant between 2020 and 2021; Blue dashed lines are statistically significant between 2020 and 2021. **A** School Closing, **B** Workplace closing, **C** Cancel public events, **D** Restrictions on gatherings, **E** Close public transport, **F** Stay at home, **G** Restrictions on internal movement, **H** International travel controls, **I** COVID-19 cases

The eight indicators from the GRT system were included to construct a model for the year 2020 and 2021, and the reference is the year 2020. Among the eight indicators from the GRT system, only Close public events, Stay at home, and Restrictions on Internal movement were statistically different between 2020 and 2021 (Fig. 3). In our primary univariate analysis of Model 1 (Table 5), the IRR was significantly different in Measles (0.82 (0.68–0.99)), Pertussis (3.16 (2.04–4.89)), Scarlet fever (2.47 (1.51–4.03)), and Seasonal influenza (3.4 (1.32–8.75)). However, this association disappeared after adjustment for eight indicators in Model 2 in multivariate analysis (Table 5).

**Table 5:** The incidence rate ratio (IRR) of seven Respiratory infectious diseases for 2021 compared with 2020

Disease	Phase 1 reported cases	Phase 2/Model 1		Phase 2/Model 2	
		IRR (95%CI)	*P-value*	IRR (95%CI)	*P-value*
Measles	1234	0.82 (0.68–0.99)	0.049	0.77 (0.41–1.45)	0.435
Tuberculosis	876,576	0.94 (0.86–1.04)	0.258	1.25 (0.8–1.94)	0.347
Pertussis	4994	3.16 (2.04–4.89)	< 0.001	1.54 (0.12–20.07)	0.748
Scarlet fever	17,206	2.47 (1.51–4.03)	0.002	5.47 (0.44–68.04)	0.211
Seasonal influenza	1,226,804	3.4 (1.32–8.75)	0.020	1.55 (0.09–27.01)	0.770
Mumps	130,911	0.97 (0.79–1.2)	0.793	0.54 (0.2–1.46)	0.249
Rubella	2839	0.78 (0.56–1.07)	0.138	1.16 (0.1–13.79)	0.907

Generalized linear models (GLM) were used for estimating the IRRs of seven Respiratory infectious diseases. Phase 1: year 2020, Phase 2: year 2021, and the reference period is 2020. The IRR of Model 1 was not adjusted; The IRR of Model 2 was adjusted by the School closing, Workplace closing, Cancel public events, Restrictions on gatherings, Close public transport, Stay at home, Restrictions on internal movement, and International travel controls. All *p*-values are two-sided and not adjusted for multiple comparisons

## Discussion

There were 25,980,811 incident cases included in our research, with a yearly average of 2,165,068 cases which was a huge burden on the public health of China. Among the 40 notifiable reported infectious diseases, seven respiratory diseases accounted for 31.03% of all the diseases from 2010 to 2021.With the outbreak of COVID-19, China has implemented a lot of personal protective measures such as hand washing, wearing masks, and social distance. Social measures such as workplace closing, school closing, canceling public events, stay-at-home requirements, restrictions on gathering size, closing public transport, restrictions on internal movement, and international travel controls, could reduce the prevalence of respiratory diseases.

Our study demonstrated the incidence of the seven respiratory diseases decreased rapidly during the COVID-19 era. Take Seasonal influenza as an example, it has 3,507,306 cases in 2019 and 986,543 cases in January 2020, but the remaining 2020 and 2021 were only 894,917. Similar results were also found in previous research [10, 11, 13] on China, and other countries such as Australia [24], America [25], and New Zealand [26]. We constructed the ARIMA model for the seven respiratory diseases, and the models fitted well in the year 2010–2019 but fitted badly in the year 2020–2021. The predicted incidence for the year 2020–2021 was higher than the actual data (Fig. 2). Tuberculosis has the smallest relative reduction, but Seasonal influenza and Rubella have the highest relative reduction.

Two reasons can be explained by the decline of respiratory diseases. Firstly, under strict control measures, the route of disease transmission has been broken, making it difficult for susceptible people to be exposed to pathogens. Chinese researchers [27] found travel out of Wuhan after the Chinese Lunar New Year averaged 6.7 million people in 2017 and 2018, but the travel ban prevented almost all of that movement in 2020. With strict NPIs, such as workplace closing, school closing, and public transport closing, there were hundreds of million people isolated at home without contact with the outside. Secondly, the NPIs to mitigate COVID-19 could affect the testing and treatment of respiratory diseases. We found the more stringent the control measures, the more declines in reported cases, especially for February 2020 with the highest scores of eight indicators and lowest incidence for the seven respiratory diseases. A study [28] showed that 26.9% of Chinese tuberculosis patients had postponed or missed their follow-up examinations due to travel restrictions and fear of COVID-19. In America, researchers [29] found a 61% decrease in the number of specimens submitted and a 98% decrease in influenza activity determined by the percentage of submitted specimens testing positive.

As the strictest NPIs implemented, China initiated a level-1 response to the COVID-19 pandemic, and the number of newly COVID-19 patients was decreasing quickly around the country [30]. With the decreasing COVID-19, all provinces downgraded public health response to level 2 or level 3, but stringent NPIs still remained in China, e.g., routine temperature monitoring, social distancing, and limiting businesses. When schools reopened on Sep 1, 2020, businesses and entertainment activities resumed nationwide marking the end of strict NPIs. Study [31] found that working resuming has little effect on the COVID-19 resurgence. We extracted eight indicators from the GRT system to quantify the effect of NPIs on respiratory infectious diseases. The correlation analysis showed all the eight control measures had negative associations with the seven diseases.

The IRR of the year 2020 and 2021 were all significantly below 1 compared with the year 2019, which suggested the prevalence of seven respiratory diseases reduced during the COVID-19 era compared with the year 2019 before the outbreak of COVID-19. Many studies have demonstrated the decreasing trend in the first year of COVID-19 [6, 11, 17, 26, 29, 32], but our study showed that the longer-term second year of COVID-19 was also decreasing. We then constructed a GLM with eight indicators for the year 2020 and 2021 corresponding to the COVID-19 pandemic and found IRR was not significant after adjusting for the eight control measures. Similar outcomes were also seen in other research, the incidence increased with the relaxation of NPIs. Wenping et al. showed the 2020–2021 influenza season did not occur, but circulations of the respiratory syncytial virus, parainfluenza, and seasonal coronavirus have returned since spring 2021 after the easing of NPIs [32]. Research conducted in Shanghai [33] found the positive rates of respiratory syncytial virus and enteric adenoviruses increased as the NPIs were relaxed in 2021. In our study, we did see a rising trend for the seven respiratory diseases at the end of 2021 when the NPIs relaxed in China (Fig. 1, Table 5), but the rising trend disappeared after adjusting for the eight indicators.

There are two main differences between our research and previous studies. Firstly, many studies [10, 12, 13, 17, 34] only evaluate infectious diseases in 2020, while our research has studied the data in 2021; secondly, methods of evaluating NPIs. Many studies [11, 13, 34] evaluated NPIs mainly by classifying NPIs at some large social points, while we evaluate NPIs by some quantitative indicators, which can more objectively reflect the impact of NPIs on diseases. Our research shows that the implementation of NPIs can effectively block the spread of respiratory infectious diseases. But changes in the reported cases of chronic respiratory infectious diseases (such as tuberculosis) may be more related to insufficient reporting than to NPI, and it is necessary to closely monitor the potential increase in disease progress and mortality due to delayed diagnosis and treatment during the pandemic [35]. The travel and assembly bans and other highly destructive NPIs cannot be used as long-term solutions, because they cause great harm to economic and social activities. Whether to restore the normal operation of the whole society or to maintain NPIs to stop the disease, many countries in the world have given different answers. It is hard to say that such a practice is necessarily correct, but combining less destructive NPI with effective vaccines and treatment schemes, such as social distancing and large indoor gatherings and wearing masks for public transport, may be a better solution.

Our study has some limitations. First, the monthly data we collected from the government website was originally from the China Information System for Disease Control and Prevention, which inevitably had a bias. Patients with respiratory diseases can’t be diagnosed by medical services which causes the missing reports, and thus the actual cases could be underestimated. Second, many variables could affect the prevalence of respiratory diseases, such as population vaccination, climate change, and virus variation, but we only included the eight indicators of NPIs. More variables should be included to specify the impact of NPIs in the future. Third, the monthly data was the national data, but not specific to provinces or cities, which may neglect the spatial heterogeneity of disease patterns. Finally, it seems almost impossible to independently assess the relationship between each NPIs and its outcome. One approach to consider would be to assess the impact in a bundled way like a sum. However, all the NPIs implemented in China represented different interventions, and the sum for the NPIs was too crude. Future research should find a better way to bundle the NPIs. In conclusion, our study demonstrated the incidence of the seven respiratory diseases decreased rapidly during the COVID-19 pandemic in 2020 and 2021. At the end of 2021, we did see a rising trend for the seven respiratory diseases compared to the year 2020 when the NPIs relaxed in China, but the rising trend was not significant after adjusting for the NPIs indicators. Our study showed that NPIs have an effect on respiratory diseases, but Relaxation of NPIs might lead to the resurgence of respiratory diseases.

## Supplementary Information

**Additional file 1: Supplementary Table S1.** Each month’s Relative reduction of seven respiratory diseases in 2020 and 2021. **Supplementary Table S2.** Sensitivity analysis for model-estimated incidence rate ratio (IRR) of seven Respiratory infectious diseases.

**Additional file 2: Supplementary Fig. 1.** The epidemic process of COVID-19 and nonpharmaceutical interventions from 2020 to 2021 in China.

## Data Availability

The R script can be found online (https://osf.io/hvdjn/), and further inquiries can be directed to the corresponding authors. The data is taken from publicly available sources which can be found mainly on the following websites: 1. website of the National Health Commission (http://www.nhc.gov.cn/jkj/s2907/new_list.shtml); 2. website of University of Oxford. COVID-19 Government Response Tracker (https://www.bsg.ox.ac.uk/research/research-projects/covid-19-government-response-tracker).

## Abbreviations

COVID-19: Coronavirus disease 2019
NPIs: Nonpharmaceutical interventions
ARIMA: Autoregressive integrated moving average
GLM: Generalized linear model
IRRs: Incidence rate ratios
GRT: Government Response Tracker
RMSE: Root mean square error
MAPE: Mean absolute percentage error
AIC: Akaike’s information criterion
BIC: Bayesian information criterion
