# Supplementary material for: Trends in respiratory diseases before and after the COVID-19 pandemic in China from 2010 to 2021
Source: BMC Public Health. 2023 Feb 1;23:217. doi: 10.1186/s12889-023-15081-4 (PMC9889952; doi:10.1186/s12889-023-15081-4)
Supplement: Supplementary file 1 — Additional file 1: Supplementary Table S1. Each month’s Relative reduction of seven respiratory diseases in 2020 and 2021. Supplementary Table S2. Sensitivity analysis for model-estimated incidence rate ratio (IRR) of seven Respiratory infectious diseases. [file 12889_2023_15081_MOESM1_ESM.docx]

Supplementary Table S1. Each month’s Relative reduction of seven respiratory diseases in 2020 and 2021.

| Time | Relative reduction (%) | | | | | | |
| --- | --- | --- | --- | --- | --- | --- | --- |
|  | Measles | Tuberculosis | Pertussis | Scarlet fever | Seasonal influenza | Mumps | Rubella |
| 2020-01 | 5.6 | 13.3 | 10.5 | 30.9 | 34.9 | 25.8 | 32.4 |
| 2020-02 | 67.6 | 33.5 | 49.4 | 86 | 95.5 | 43 | 81.3 |
| 2020-03 | 74.7 | 23.3 | 68.2 | 92.4 | 98.4 | 71.4 | 95.3 |
| 2020-04 | 85.6 | 7.8 | 82.3 | 94.5 | 98.9 | 79.7 | 98.7 |
| 2020-05 | 83.8 | 8 | 91.2 | 95.3 | 98.7 | 75.8 | 99.2 |
| 2020-06 | 74.8 | 2.8 | 94.9 | 94.4 | 98.7 | 69.6 | 98.5 |
| 2020-07 | 65.7 | 4 | 96.4 | 87.9 | 98.9 | 66.9 | 97.5 |
| 2020-08 | 61.1 | 8 | 96.8 | 77.1 | 98.9 | 57.5 | 94 |
| 2020-09 | 53.9 | 4 | 94 | 77.9 | 98.4 | 35.4 | 90.6 |
| 2020-10 | 44.4 | 5.9 | 86.3 | 81.2 | 98.2 | 42.6 | 88.2 |
| 2020-11 | 55 | 6.5 | 82.6 | 80.9 | 98.2 | 44.9 | 82.2 |
| 2020-12 | 61.6 | 12.4 | 79.4 | 78.9 | 98.8 | 60.9 | 95.5 |
| 2021-01 | 75.6 | 14.6 | 84.1 | 72.6 | 99 | 72 | 94.5 |
| 2021-02 | 83.7 | 16.7 | 92.2 | 72.4 | 99.5 | 73.3 | 97.9 |
| 2021-03 | 72.2 | 14.2 | 92 | 68.7 | 99.2 | 56.7 | 97.8 |
| 2021-04 | 80.7 | 10.9 | 88.7 | 66 | 98.4 | 62.7 | 98.4 |
| 2021-05 | 79.6 | 14.8 | 86.5 | 64.3 | 97.9 | 69.3 | 97.6 |
| 2021-06 | 78.3 | 12.9 | 83.6 | 65 | 98 | 70.3 | 98 |
| 2021-07 | 74 | 8.4 | 77.6 | 64.4 | 98.7 | 72.2 | 96.8 |
| 2021-08 | 77.8 | 15.3 | 72.2 | 66.7 | 98.9 | 63.5 | 96.8 |
| 2021-09 | 66.9 | 10.2 | 67.3 | 71.8 | 98.2 | 41.2 | 94.9 |
| 2021-10 | 62.8 | 11 | 58 | 74.2 | 97.3 | 51.8 | 94.8 |
| 2021-11 | 61.1 | 13.5 | 31.8 | 75.7 | 94.4 | 57.9 | 94.3 |
| 2021-12 | 63 | 11.6 | -56.1 | 71.6 | 89.2 | 64.4 | 94 |

Relative reduction (%) = 100% * (number of expected cases – number of observed cases)/number of expected cases.

Supplementary Table S2. Sensitivity analysis for model-estimated incidence rate ratio (IRR) of seven Respiratory infectious diseases.

| Disease | Phase 2 (2019) reported cases | Phase 1(2010-2018) | | Phase 3 (2020) | | Phase 4 (2021) | |
| --- | --- | --- | --- | --- | --- | --- | --- |
|  |  | IRR (95%CI) | *P-value* | IRR (95%CI) | *P-value* | IRR (95%CI) | *P-value* |
| Measles | 3573 | **6.58 (3.94-10.99)** | **<0.001** | **0.15 (0.06-0.42)** | **0.002** | **0.1 (0.03-0.31)** | **0.001** |
| Tuberculosis | 1034760 | **1.22 (1.15-1.29)** | **<0.001** | **0.85 (0.78-0.92)** | **<0.001** | **0.8 (0.74-0.87)** | **<0.001** |
| Pertussis | 30727 | **0.21 (0.17-0.25)** | **<0.001** | **0.15 (0.1-0.23)** | **<0.001** | **0.38 (0.27-0.52)** | **<0.001** |
| Scarlet fever | 83028 | **0.71 (0.61-0.82)** | **<0.001** | **0.18 (0.13-0.26)** | **<0.001** | **0.36 (0.28-0.47)** | **<0.001** |
| Seasonal influenza | 3507306 | **0.08 (0.06-0.11)** | **<0.001** | **0.36 (0.24-0.52)** | **<0.001** | **0.19 (0.11-0.31)** | **<0.001** |
| Mumps | 303105 | 0.95 (0.86-1.05) | 0.342 | **0.41 (0.34-0.49)** | **<0.001** | **0.37 (0.31-0.45)** | **<0.001** |
| Rubella | 34151 | 0.53 (0.21-1.35) | 0.184 | **0.001 (0-0.11)** | **<0.001** | **0.03 (0.01-2.41)** | **<0.001** |

Generalized linear models (GLM) were used for estimating the IRRs of seven Respiratory infectious diseases. The seasonality of the reported cases was adjusted by the harmonic function method in this model. IRR < 1 with P < 0.05 indicates a significant decline in incidence rate in the year 2020 compared to the year 2019. All p-values are two-sided and not adjusted for multiple comparisons. The reference period is the year 2019. Statistically significant reductions (IRR < 1) are displayed in bolded font.
