# Supplementary figures and images for: Trends in respiratory diseases before and after the COVID-19 pandemic in China from 2010 to 2021
Source: BMC Public Health. 2023 Feb 1;23:217. doi: 10.1186/s12889-023-15081-4 (PMC9889952; doi:10.1186/s12889-023-15081-4)

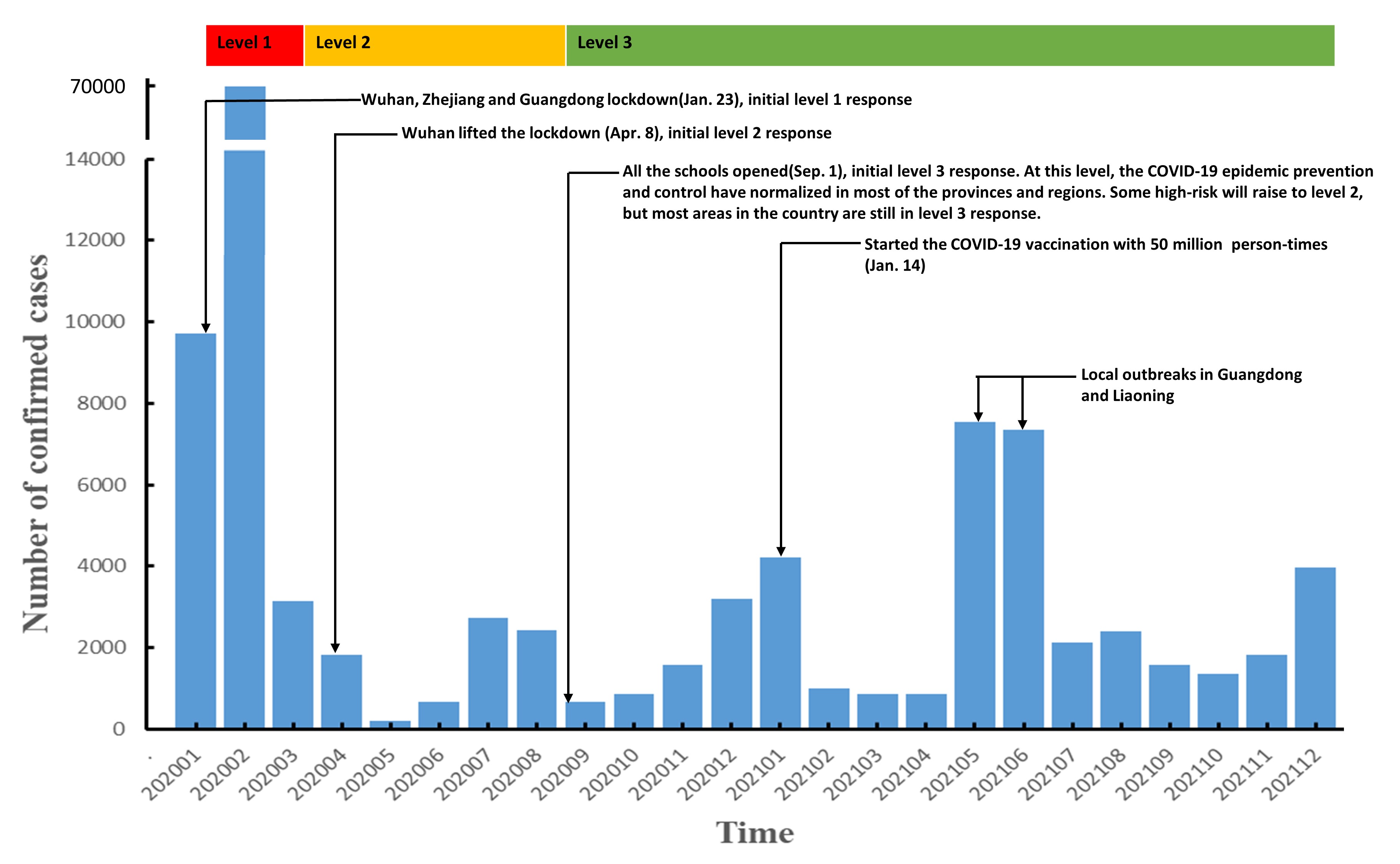

Supplement: Supplementary file 2 — Additional file 2: Supplementary Fig. 1. The epidemic process of COVID-19 and nonpharmaceutical interventions from 2020 to 2021 in China. [file 12889_2023_15081_MOESM2_ESM.jpg]
